# Dynamic Production of Soluble Extracellular Polysaccharides by *Streptococcus mutans*

**DOI:** 10.1155/2011/435830

**Published:** 2011-10-20

**Authors:** Eva-Maria Decker, Ilka Dietrich, Christian Klein, Christiane von Ohle

**Affiliations:** Department of Operative Dentistry and Periodontology, Center of Dentistry, Oral Medicine, and Maxillofacial Surgery, Eberhard-Karls University Tübingen, 72076 Tübingen, Germany

## Abstract

Caries development in the presence of *Streptococcus mutans* is associated not only with the production of extracellular water-insoluble polymers but also is based on water-soluble polysaccharides. The aim of this study was the evaluation of a novel glucan-specific Lectin assay for monitoring water-soluble EPS produced by *S. mutans* during several growth periods in different media. *S. mutans* cultures were grown for 24 h, 48 h, and 144 h in medium deficient of sucrose (A) and medium supplemented with 5% sucrose (B). Microtiter well plates were coated with cell-free supernatants followed by the addition of labeled Concanavalin-A and enzyme substrate. The substrate reactions were kinetically detected at 405 nm. The validation of the assay was performed using carbohydrates dextran, xanthan, and sucrose as reference. This new Concanavalin-A-based assay showed the highest sensitivity for dextran and revealed that the glucan production of *S. mutans* reached its maximum at 144 h in medium B according to bacterial maturation.

## 1. Introduction

The etiology of dental caries is often associated with increasing amounts of various acidogenic microorganisms like *Streptococcus mutans* which plays a keyrole in the formation of cariogenic biofilms [1]. The structural and functional properties of biofilms, like human dental plaque, are essentially determined by the presence of microbial hydrated polymers which are mainly composed of the self-produced extracellular polysaccharides (EPSs) and also of proteins, nucleic acids, phospholipids, mucosal cells, and nutrient components [1, 2]. Particularly, the EPSs produced by *S. mutans* contribute to the cariogenic potential of dental biofilms and their resistance to oral hygiene measures [3]. The EPSs of *S. mutans* during sugar exposure consist predominantly of glucose polymers (glucans) containing various proportions and branches of alpha-1.3 (water-insoluble) and alpha-1.6 (water-soluble) glucosidic linkages [4]. The sucrose and glucose metabolism of *S. mutans* involves versatile interactions and regulation of different extracellular glucosyltransferases: GtfB (water-insoluble glucan, ISG; low-molecular-weight water-soluble glucan, SG), GtfC (ISG and SG), GtfD (SG), and FtfF (water-soluble fructose polymers) [5]. Most studies addressing the microbial interrelationship of caries are focused on the relevance of water-insoluble EPSs produced by mutans streptococci and their genetic regulation [6]. Soluble carbohydrate polymers and their synthesizing enzymes have been shown to play another important role for the enhancement of caries development although the precise mechanisms are not yet clarified. Water-soluble polysaccharides may serve as a source of metabolizable carbohydrate for plaque bacteria if nutrient conditions become limited [7], and thus support cariogenic attack at the enamel surface. Water-soluble EPSs secreted into the environmental medium may participate in the matrix of dental plaque in vivo [8]. Concerning the EPSs-synthesizing enzymes, the results of Venkitaraman et al. [9] indicated a positive cooperativity of activity between GtfB and GtfD and suggested GtfD to act as an intrinsic primer for insoluble glucan synthesis by GtfB. The significance of water-soluble exopolymers could be further demonstrated by Rundegren et al. [10] revealing that the interaction of salivary components and water-soluble glucan increased the viscosity of saliva up to 65%/55% at pH 6/7. These charge-dependent interaction could influence the cohesive forces of plaque matrix. In the presence of high molecular weight glucans (soluble dextrans), bacteria were induced to aggregate and thus assist colonization [11]. The development of caries seems to require the involvement of SG and ISG synthesizing genes as shown by *S. mutans *mutants which produced significantly fewer smooth-surface lesions due to them being defective in the gtfD gene coding for SG [12]. The specificity of lectins for particular sugars is a useful tool for analysis and detection of complex glycoconjugates. The aim of this study was the establishment and application of a novel specific test system for quantitatively monitoring of water-soluble EPSs produced by *S. mutans* during different growth periods by means of the glucan-specific lectin Concanavalin A.

## 2. Materials and Methods

### 2.1. Microorganisms and Growth Conditions

*Streptococcus mutans *(ATCC 25175) were grown in Schaedler broth (Becton Dickinson) for 18 hours at 37°C. For monitoring EPSs a bacterial inoculum taken from a logarithmic phase culture of *S. mutans* was added to Schaedler broth without sucrose (medium A) and with 5% sucrose (medium B). The streptococci were grown anaerobically for 24 h, 48 h, and 144 h at 37°C. The microbial parameters total bacterial cell counts/mL (BC), percentage of vital streptococci (VS), and colony forming units/mL (CFU) grown on Schaedler agar (Becton Dickinson) were assessed at the beginning of each experiment and after each incubation period.

### 2.2. Fluorescent Staining of Microorganisms

The streptococci were stained fluorescently at each growth period by means of two DNA stainings Syto 9 and propidium iodide (Invitrogen-Molecular Probes) differentiating vital cells (green) and dead bacteria (red) by epifluorescence microscopy according to CFU production [13]. The vitality of streptococci was defined as VS (%) = 100 − proportion of dead cells.

### 2.3. Specific Carbohydrate Detection by Con A Lectin Assay

#### 2.3.1. Reference Sugars

**Figure 1:**
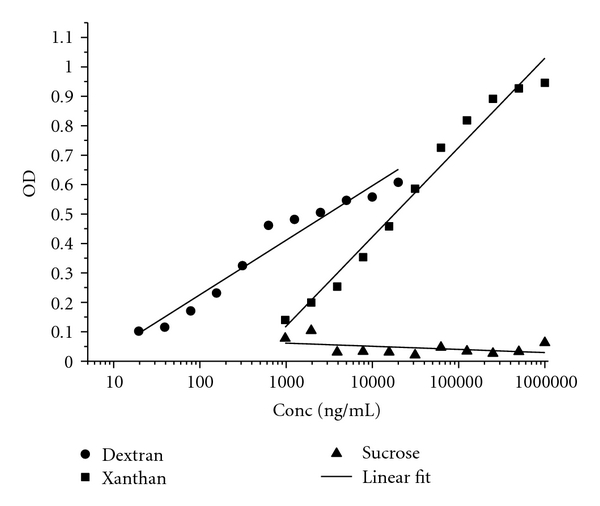
Lectin assay: standard carbohydrate calibration curves of ● dextran, ■ xanthan, ▲ sucrose, and regression lines as linear fit.

**Figure 2:**
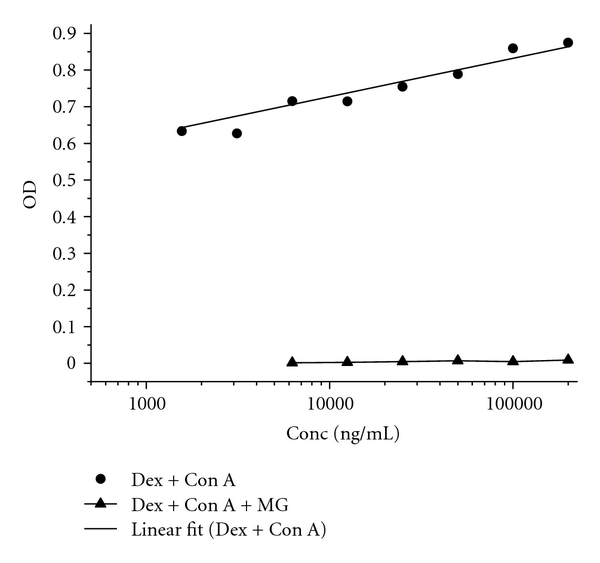
Lectin assay: ● calibration curve of dextran detected by Con A (regression line as linear fit), ▲ inhibition of Con A with *α*-methylglucoside (MG) prior to dextran detection.

**Figure 3:**
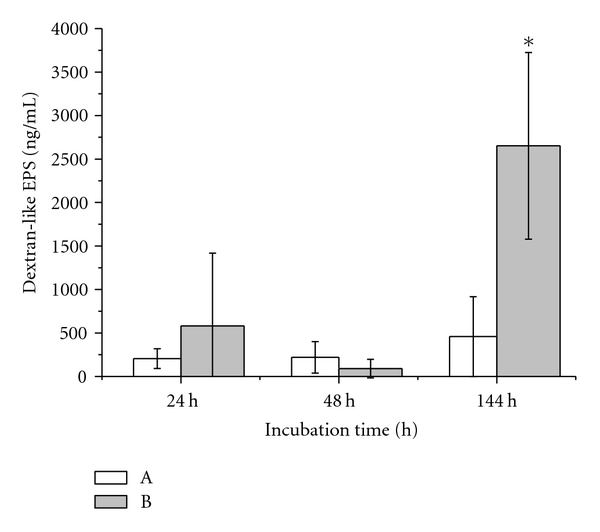
Lectin assay: mean values and mean-based 95% confidence intervals of EPSs production of *S. mutans* in the white rectangle medium A without sucrose and the grey rectangle medium B with 5% sucrose at 24 h, 48 h, and 144 h incubation time.

The glucan-specific assay was based on the sugar specificity of the lectin concanavalin A (C*anavalia ensiformis*) labelled with peroxidase (Con A) (Sigma) for glucose polymers. The lectin-based assay for the characterization of microbial polysaccharides described previously [14] was modified and optimized in the present study for the detection of soluble *S. mutans* EPSs and corresponding reference sugars. In the present test system, a working solution of 20 *μ*g/mL Con A in physiological saline was prepared. The enzyme-linked Lectin assay was evaluated with three carbohydrate standards: dextran representing the sugar equivalent to the EPSs matrix of *S. mutans*, xanthan, a heterogenous polysaccharide composed of glucose, mannose, and glucuronic acid, and sucrose, a disaccharide containing glucose and fructose. 100 *μ*L aliquots of serial dilutions 1 : 2 diluting stock solutions of dextran (20 *μ*g/mL) and of xanthan and sucrose (1 mg/mL) with physiological saline (NaCl) as well as assay controls were added to 96-well microtiter plates in eleven dilution steps and coated for 20 h. Subsequently, wells were washed with 200 *μ*L sterile water. 100 *μ*L of Con A working solution was added to each well and incubated for one hour. After three washing procedures with each 200 *μ*L PBS with 0.05% Tween, 100 *μ*L of the freshly prepared peroxidase substrate ABTS (Sigma) with hydrogen peroxide were added. At 405 nm, kinetic measures of optical density were performed every 5 minutes during 60 min. The Figures 1–3 correspond to the data derived from the kinetic substrate incubation for 45 min. Six calibration curves were created for each standard carbohydrate. The concentrations of the standard sugars were log_10_ transformed followed by a logarithmic regression for curve fitting.

#### 2.3.2. EPSs of *S. mutans* Cultures

The EPSs synthesized by *S. mutans* were monitored under two nutrient conditions: without sucrose (A) and with 5% sucrose supplement (B). The cell-free supernatants of the bacterial cultures were coated on microtiter plates according to the conditions described in Section 2.4.1. After a wash step, the plates were incubated with Con A. The colour development of the peroxidase reaction was induced by addition of the enzyme substrate ABTS and hydrogen peroxide. The optical density was monitored kinetically every five minutes for one hour by means of an Elisa reader at 405 nm. Six bacterial experiments at any growth condition (24 h, 48 h, and 144 h) were conducted. In addition, sterile media A and B were assayed for Con A detection by Lectin assay. Any experimental approach of Con A-based Lectin assay detecting sterile or grown nutrient solutions was completed by using internal standard of dextran reference.

#### 2.3.3. Inhibition Assay of Con A

The specific binding of Con A to glucan was verified by inhibiting the active binding site of Con A by adding *α*-methylglucoside (MG) to make sure that the assay signals resulted from glucan recognition and not from unspecific binding in mutans cultures [15]. Reference carbohydrates and supernatants of *S. mutans* cultures were coated in microtiter plates as described. Con A was preincubated with 200 mM *α*-methylglucoside or NaCl as negative control for 30 min. Subsequently, Con A solutions were applied for Lectin assay according to the procedures as per description. 

### 2.4. Chemical Total Carbohydrate Detection by Dubois Assay

#### 2.4.1. Reference Sugars

Mono-, oligo-, and polysaccharides give a sensitive colour reaction after treatment with phenol and concentrated sulphuric acid. The colorimetric method for determination of sugars [16] was modified using submicroamounts of reagents. The standard sugar concentrations (dextran, xanthan, and sucrose) used for calibration of the method were 25, 50, 75, 100, 200, 300, 400, 500, and 600 *μ*g/mL. 30 *μ*L sample or negative control were mixed with 30 *μ*L 5% phenol and 150 *μ*L concentrated sulphuric acid. After 30 min, the optical density (OD) of samples was evaluated at 490 nm. 

#### 2.4.2. Total Carbohydrate Concentration of  *S. mutans* Cultures

Sugar concentrations of supernatants of *S. mutans* cultures were analyzed chemically after 24 h, 48 h, and 144 h growth time in Schaedler media A and B.

### 2.5. Statistical Analysis

All experiments were performed as six independent experiments in triplicates (Lectin assay, Dubois assay) including an internal dextran standard in each series. A logarithmic transformation (base 10) was done for BC and CFU. Statistical analysis of data was performed using mean-based 95% confidence intervals and Kruskal-Wallis test at significance level of *α* = 0.05.

## 3. Results

The Con A-based Lectin assay was evaluated with the reference carbohydrates dextran, xanthan, and sucrose as illustrated in Figure 1. Con A showed the highest sensitivity for the high-molecular dextran with a detection limit of 10 ng/mL within the linear measuring range. The detection limit for xanthan was about 500 ng/mL revealed by Con A. Sucrose did not induce concentration-dependent specific binding of Con A. 

The specificity of the carbohydrate detection of Con A for glucose polymers was verified by inhibition of the carbohydrate-binding sites of the lectin molecule using *α*-methylglucoside (Figure 2). The supernatants of *S. mutans* cultures grown for 24 h, 48 h, and 144 h in media A and B were monitored for EPSs production by application of Con A-based Lectin assay (Figure 3). Some differences of the 95% confidence intervals due to EPSs profiles could be observed concerning media A and B. Whereas EPSs in medium A could be detected at all incubation time points, the corresponding EPSs signals detected from medium B appeared predominantly at 24 h and 144 h incubation times. The dextran-like EPSs peak in 144 h B exceeded the glucan signal produced in A. Considering the dilution profiles of 24 h, 48 h, and 144 h EPSs production, an increase of dextran peaks at longer incubation times from 24 h to 144 h was evident for both media. Quantitative EPSs calculations were performed by correlating the data of dextran-like EPSs peaks in mutans cultures to those of fitted dextran calibration curves (Figure 3).

Comparing EPSs profiles in media A and B the presence of sucrose in growth medium seemed to increase the glucan production by *S. mutans* cells at 24 h incubation time. At 48 h incubation time, the EPSs concentration produced in A remained constant in contrast to the decreasing EPSs concentration in B. After 144 h incubation the EPSs concentrations rose in both nutrient solutions. Whereas the EPSs increase produced at 144 h in sucrose-deficient medium was about twice the production at 24 h and 48 h, the dextran-like EPSs in sucrose-supplemented medium showed at this time point a statistically significant increase which was about 5.8 times higher than the EPSs maximum produced after 144 h in A and about five times higher than the EPSs production at 24 h in B. The quantitative differences of EPSs contents were independent from the total cell numbers showing constant values over time.

**Figure 4:**
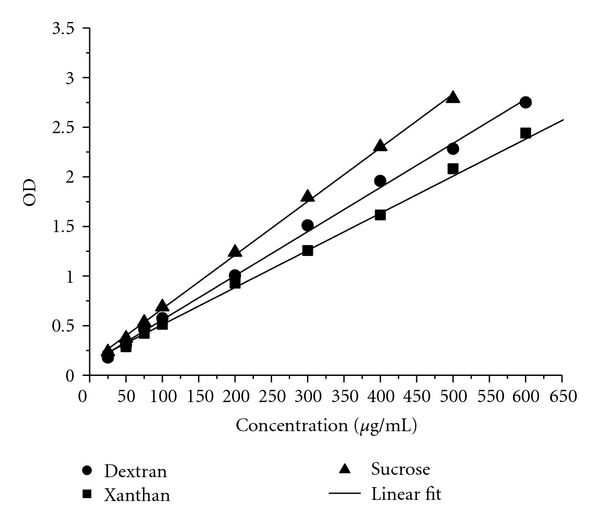
Dubois assay: standard carbohydrate calibration curves of ● dextran, ■ xanthan, ▲ sucrose, and regression lines as linear fit.

**Figure 5:**
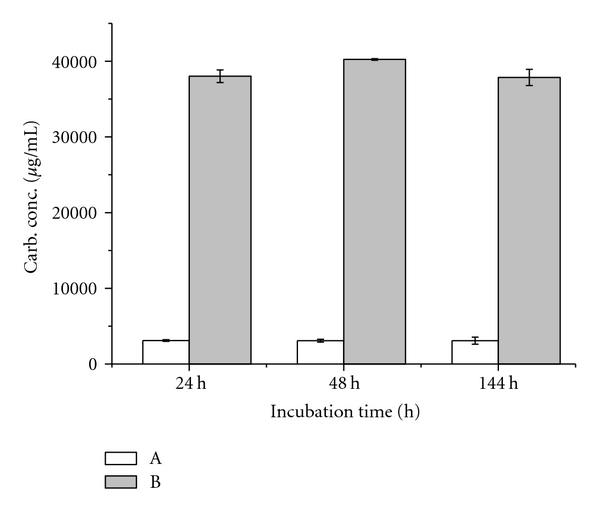
Dubois assay: mean values and mean-based 95% confidence intervals of carbohydrate concentration in *S. mutans* cultures of the white rectangle medium A without sucrose and the grey rectangle medium B with 5% sucrose at 24 h, 48 h, and 144 h incubation time.

The chemical detection of total carbohydrates using Dubois assay showed a similar detection limit for dextran, xanthan, and sucrose of about 20 *μ*g/mL (Figure 4). Carbohydrate monitoring at 24 h, 48 h, and 144 h was similar for medium A and for medium B, respectively as shown by 95% confidence intervals (Figure 5). Collectively, the sugar content of medium B was about twelve times higher on average than that of medium A. 

**Figure 6:**
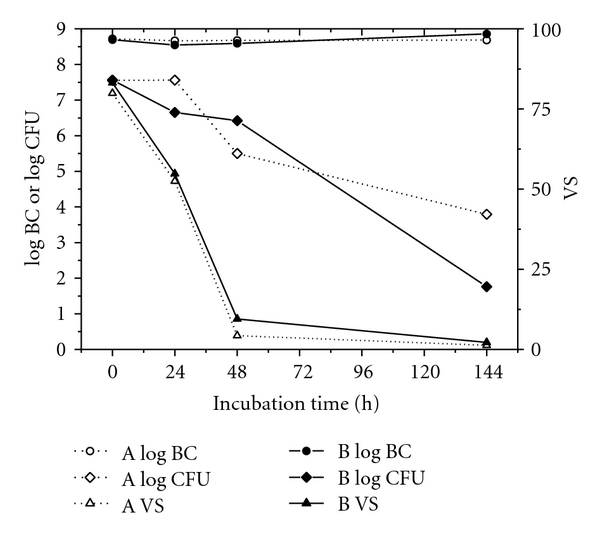
Microbiological parameters of *S. mutans* cultures in medium A without sucrose (open symbols) and medium B with 5% sucrose (filled symbols): ○/● log BC, *◊*/*◆* log CFU, ∆/▲ vitality (VS) at 24 h, 48 h, and 144 h incubation time.

The microbiological growth parameters VS, log BC, and log CFU showed a similar profile for *S. mutans* growth in media A und B (Figure 6). Whereas the total bacterial cell counts remained constant, bacterial vitality decreased at 48 h near to zero. The CFU values of streptococci grown in sucrose-enriched medium showed a stronger decline of two log_10_ units compared to *S. mutans* cells cultured in sucrose deficient medium. Morphologically, mutans streptococci grown as distinctive microcolonies only in the presence of sucrose-supplemented medium. 

## 4. Discussion

The objective of this study was the development of a specific test system feasible for monitoring the dynamic production of water-soluble dextran-like EPSs of mutans streptococci under different nutrient conditions and growth time points. The performance of the Lectin assay with standard carbohydrates (dextran and xanthan), sterile media samples, and inhibition test with *α*-methylglucoside verified the specific validity of the test system for *S. mutans*-associated soluble glucan exopolymer detection. The statistically significant increase of streptococcal soluble EPSs in the medium supplemented with sucrose at a late growth phase was evident. The time-dependent monitoring of dextran-like EPSs revealed a dynamic cumulation of polymers during longer streptococcal incubation periods in both growth media with and without supplement of sucrose. It is clinically relevant that sucrose has a proven key role in caries activity serving as fermentable carbohydrate source or inducing carbohydrate polymerization [3, 17]. The slime character of these glucan-rich sugar polymers mediates the central impact of glucan on sucrose-dependent bacterial adhesion to tooth surfaces and the correlation between sucrose exposition and increased caries rates [18] although other factors additionally may affect cariogenicity [19]. Furthermore, bacterial ability of resistance to antimicrobials seems to be linked to the glucan production [17]. 

Concerning the different availability of carbohydrates, fast utilizable monosaccharides (e.g., 0.58% glucose in medium A and B) are preferentially metabolized by microorganisms. In the present study, the exposure of* S. mutans* cells to sucrose might have induced a shift in the carbohydrate metabolism involving downregulation of glucose-related enzymes based on depletion, increase of sucrose utilization and in the further course the stimulation of carbohydrate polymerizing activity over time resulting in the water-soluble dextran-like EPSs maximum at 144 h. Only few comparable studies are available concerning research of SG exopolymers. The study of Shemesh et al. [20] revealed that mRNA expression of the glucosyltransferase-encoding genes, gtfB, gtfC, and gtfD, of *S. mutans* is carbohydrate-regulated and sucrose stimulated the synthesis of glucosyltransferases GtfB and GtfC more pronounced in the early exponential phase. With regard to the expression of SG synthesizing GtfD the presence of sucrose induced only low enzyme level in the early and late exponential phase [21]. In a planktonic environment strong metabolic changes of transcriptional and translational products are involved during microbial maturation from the early exponential growth phase to the late stationary phase. Possible conflicting results indicate that the carbohydrate metabolism depends substantially on environmental conditions such as growth media, carbohydrate content, bacterial strains, carbohydrate uptake, and laboratory conditions. Recently, it has been shown that *S. mutans* cells are able to alter their pathogenic potential and metabolism dramatically in response to exposure to oxygen and depending on the maturity of bacteria [22]. Comparing the present data of Lectin assay and chemical analysis, the detection limit of carbohydrates monitored by Con A assay was hundredfold lower than those analyzed by means of Dubois assay. This chemical analysis confirms the differences of carbohydrate content between media A and B, whereas differences during the growth periods of 24 h, 48 h, and 144 h could not be detected.

Contrary to the time response of the EPSs cumulation during 24 h to 144 h incubation was the bacterial decrease of vitality and capaciticiy of colony growth under both nutrient conditions. 

It is conceivable that the streptococci lost their ability of reproduction and metabolic activity under unfavourable conditions but induced a cumulation of extracellular polymers as part of their survival strategies. The observation in the present study that mutans streptococci grown as distinctive microcolonies only in the presence of sucrose was in agreement with studies of Renye et al. [23].

In conclusion, the Con A Lectin assay seemed to be adequate for the detection of dextran-like soluble EPSs of *S. mutans* during different growth times within the conditions of the present study. The EPSs production increased after sucrose exposition and during microbial maturation with extended incubation time (144 h). Further studies are needed to substantiate potential clinical application. Monitoring soluble EPSs in the saliva of caries-risk patients might serve as valuable tool in addition to clinical parameters.
